# Redox Status of Pregnant Ewes after Vaccination against Clostridial Diseases

**DOI:** 10.3390/vaccines10060898

**Published:** 2022-06-05

**Authors:** Evaggelos-Georgios Stampinas, Efterpi Bouroutzika, Panagiotis D. Katsoulos, Georgios Valiakos, Ekaterini K. Theodosiadou, Labrini V. Athanasiou, Sotiria Makri, Demetrios Kouretas, Irene Valasi

**Affiliations:** 1 Laboratory of Physiology, Faculty of Veterinary Science, University of Thessaly, 43131 Karditsa, Greece; estampinas@vet.uth.gr (E.-G.S.); bouroutz@vet.uth.gr (E.B.); etheodosiadou@vet.uth.gr (E.K.T.); evalasi@vet.uth.gr (I.V.); 2 Clinic of Farm Animals, School of Veterinary Medicine, Aristotle University of Thessaloniki, 54124 Thessaloniki, Greece; 3 Laboratory of Microbiology and Parasitology, Faculty of Veterinary Science, University of Thessaly, 43131 Karditsa, Greece; georgevaliakos@vet.uth.gr; 4 Department of Medicine, Faculty of Veterinary Science, University of Thessaly, 43131 Karditsa, Greece; lathan@vet.uth.gr; 5 Department of Biochemistry and Biotechnology, University of Thessaly, Viopolis, Mezourlo, 41500 Larissa, Greece; sotirina_m@hotmail.com (S.M.); dkouret@uth.gr (D.K.)

**Keywords:** ewes, redox status, polyvalent clostridial vaccine, pregnancy, vitamin E, selenium

## Abstract

The redox status shortly after the vaccination of pregnant ewes is rather unexploited. Thus, the present study was designed to evaluate the fluctuation of redox status after the administration of the annual booster dose of a polyvalent clostridial vaccine in pregnant ewes, 3 to 4 weeks before lambing, with or without a simultaneous injection of Vit E/Se. In total, 24 pregnant Lacaune ewes 3–4 weeks before lambing were randomly allocated into four equal groups: the V (vaccinated with a polyvalent clostridial vaccine), VE (vaccinated and injected IM with Vit E/Se), E (injected IM with Vit E and Se), and C (neither vaccinated nor injected with Vit E/Se). The study period lasted for 21 days, starting on the day of administration. Four redox biomarkers, the antioxidant capacity (TAC), the thiobarbituric acid reactive substances (TBARS), the reduced glutathione (GSH), and the catalase (CAT) were evaluated in blood samples collected from all ewes before the injections (0 h) and then at 12 (12 h), 24 (D1), and 48 h (D2), and thereafter on days 4 (D4), 6 (D6), 10 (D10), 14 (D14), and 21 (D21). The results reveal that the TAC was the only biomarker evaluated that was significantly affected by group and significantly lower in vaccinated animals (V and VE groups) compared to non-vaccinated (E and C groups). The absence of an increase in the TBARS values after vaccination in groups V and VE indicates the absence of significant oxidative stress. Overall, it can be assumed that annual booster immunizations against clostridial diseases do not impose acute oxidative stress on pregnant ewes in the last month of pregnancy.

## 1. Introduction

Free radicals play a critical role in various physiological processes, such as immunization and pregnancy outcomes. The oxidant/antioxidant balance greatly affects the normal function of immune cells by maintaining cellular integrity and functionality, as well as the cellular protein and nucleic acid content. However, the great sensitivity of immune cells to oxidative stress is partly due to the high percentage of polyunsaturated fatty acids in cellular membranes, which are highly susceptible to lipid peroxidation. During the immune response induced by vaccination, macrophages and dendritic cells are recruited and activated for antigen processing, which leads to the generation of reactive oxygen species (ROS) and results in inflammatory sequelae [1]. Thus, the presence of antioxidants in immune cells is of great importance for diminishing the oxidative stress therein and for ensuring their normal activity.

Oxidative stress is defined as the pro-oxidant/antioxidant imbalance in favor of oxidants, leading to the disruption of redox signaling and control and/or molecular damage [2]. The antioxidant defense system comprises enzymatic and non-enzymatic antioxidant mechanisms. The enzymatic mechanisms are distinguished as primary, such as glutathione peroxidase (GPx), catalase (CAT), and superoxide dismutase (SOD), and secondary, such as the glutathione reductase (GR) [3], whereas the non-enzymatic include glutathione (GSH) [4] and a number of vitamins and trace elements, such as vitamin E (Vit E), vitamin C (Vit C), selenium (Se), and others [5]. Interactions between the enzymatic and non-enzymatic antioxidant mechanisms further affect the redox status and immune function [6] Specifically, Vit E acts as a scavenger and transforms into a stable radical, which is restored as Vit E in the cell by Vit C, GSH, ubiquinone, and other pathways [7], while Se is a component of selenoproteins, such as the GPx enzyme [6]. The metabolic function of Se is intimately linked to Vit E, and both compounds protect the cellular membranes from oxidative degeneration [6,8].

Specific vaccination programs are applied in sheep flocks as a health management practice, including a vaccination schedule for clostridial diseases [9]. Enteropathogenic bacteria, such as *Clostridium perfringens*, produce several virulence factors that can cause enterotoxaemia both in lambs and sheep and are associated with Type A and Type B of *C. perfingens*. Type B isolates especially often cause fatal hemorrhagic dysentery in sheep and increase the lambs’ death rate in a flock [10]. For this reason, the active immunization of young lambs against these bacteria is recommended after the age of 6 to 8 weeks. Thereafter, booster immunizations, at least annually, should be applied [9]. Concerning the high vulnerability of neonates to these diseases, the vaccination of pregnant ewes against clostridiosis 3 to 4 weeks before lambing consists of a preventive management practice, given that newborn lambs can be protected via the absorption of antibodies ingested with colostrum [11]. 

However, during pregnancy in sheep, both the maternal and fetal organisms are exposed to oxidative stress caused by the increased amount of ROS [12]. The peri-parturient period is especially characterized by a high generation of these compounds because of increased metabolic demands related to near-term pregnancy, parturition, lactogenesis, and the onset of lactation. The abundant ROSs lead to a transient impaired immune response, which is further induced by the increased cortisol levels before parturition [13]. Hence, the increased ROSs may interfere with any active immunization during this vulnerable period. So far, some studies have been made for understanding any relationship or interaction between the redox status and the immune response induced by vaccination in animals, with various outcomes. The administration of Rev.1 conjunctival *Brucella melitensis* vaccine caused a decrease in the total antioxidant status one month after the vaccination of 5-month-old female sheep [14], and an increase in serum glutathione peroxidase activity and malondialdehyde levels by the fourth week after the vaccination of pregnant ewes, depending on the dose and route of administration [15]. The redox status was also evaluated in a pilot study in cattle, which provided evidence that the vaccines that induce lower oxidative stress induce a higher production of antigen-specific antibodies [16]. Based on this fact, many researchers have evaluated the impact of the supplementation of antioxidants, such as Vit E and/or Se, on the immune response of pregnant or non-pregnant sheep after vaccination [13,17,18,19,20,21,22]. Nevertheless, there is still limited and quite loose knowledge about the effect of vaccination on the redox status of pregnant ewes. Thus, the objective of the present study was the evaluation of the fluctuation of redox status just after the administration of the annual booster dose of a polyvalent clostridial vaccine in pregnant ewes, 3 to 4 weeks before lambing, with or without a simultaneous injection of Vit E/Se.

## 2. Materials and Methods

### 2.1. Animals and Experimental Design

In total, 24 2–4-year-old Lacaune ewes, 3–4 weeks before the expected day of lambing, were used for the purpose of the study. According to the farm vaccination protocol against clostridial diseases, all replacement animals receive a first vaccine dose at the age of 2 months, the second 3–4 weeks afterwards, and then an annual booster dose about one month before the expected day of lambing. For synchronizing the time of lambing, ewes were treated with intravaginal progestogen sponges and an intramuscular eCG administration at the removal of the sponges. Pregnancy was confirmed by ultrasonography (U/S) 50 days after the sponges’ removal and was repeated before the initiation of the experiment for the confirmation of pregnancy maintenance and the presence of live fetuses. Anthelmintic treatment (10 mg/kg BW/ewe Albendazole oral 10%; Provet) was applied one month before the onset of the study, and no other vaccine or treatment was administered for at least 6 months. 

Proportionally by age (mean age of 3 years in all groups), the ewes were randomly allocated as follows: the V group (*n* = 6) consisted of ewes that received an annual booster dose of a polyvalent clostridial vaccine (dose rate: 2 ml SC; Panclostil, Ceva Hellas S.A.); the VE group (*n* = 6) consisted of those that were vaccinated and were intramuscularly injected with a solution of Vit E/Se (dose rate: 30 IU/kg BW; Vitamin E-Selen, MSD Animal Health Hellas) [17]; the E group (*n* = 6) consisted of those that were intramuscularly injected with a solution of Vit E/Se; and the C group (*n* = 6) consisted of those that did not receive either the vaccine or Vit E and Se, and served as controls. The dosage of Vit E was based on previously tested protocol [17]. In addition to the aforementioned interventions, the ewes of group V received an intramuscular placebo dose of normal saline (NS 0.9%) at the same volume of the Vit E/Se. Those of group E received a subcutaneous placebo administration of 2 ml of NS 0.9%, and the ewes of group C had both an intramuscular and a subcutaneous administration of NS 0.9% at the volume previously mentioned.

**Figure 1:**
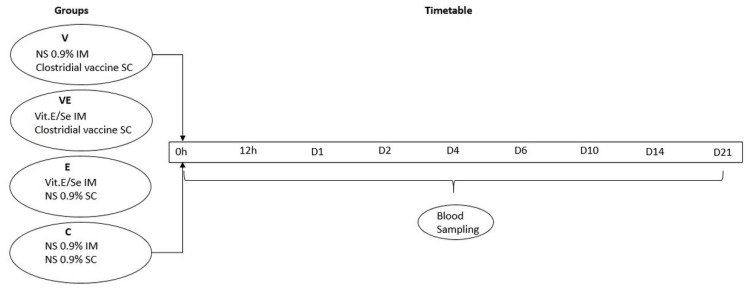
Experimental overview of the study.

The study period started on the day of vaccine and Vit E/Se administration (day 0) and lasted until day 21 after administration (Figure 1). Throughout this period, all animals were housed together in a single straw-bedded pen and received the same rations. The rations were offered twice daily and consisted of 0.5 kg of a commercial concentrate feed for dry ewes in mash form, plus 1.2 kg of alfalfa hay per ewe. In addition, barley straw was provided thrice daily for ad libitum consumption, whilst water was available ad libitum. Selenium and Vit E were included at rates of 0.25 mg/kg and 40 mg/kg, respectively, in the commercial concentrate feed as the management practice according to NRC [23] and Dønnem et al. [24].

Clinical monitoring of the animals was performed for the detection of any general or local adverse reactions at the injection site. During the 4 days following vaccination, the body temperatures of the animals were measured daily by a rectal digital thermometer.

### 2.2. Blood Sampling

Blood samples were collected from all ewes by jugular venipuncture using vacutainer tubes with EDTA before the injections (0 h) and then at 12 h (12 h), 24 (D1), and 48 h (D2), and thereafter on days 4 (D4), 6 (D6), 10 (D10), 14 (D14), and 21 (D21) post-injections. 

### 2.3. Determination of Redox Status Biomarkers

The blood samples were appropriately prepared after collection and then stored at −80 °C. All measurements were performed within 3 months of collection.

Four redox biomarkers, namely the total antioxidant capacity (TAC) as a crude index of the antioxidant potential of the examined biological fluid, thiobarbituric acid reactive substances (TBARS) as a biomarker of lipid peroxidation, reduced glutathione (GSH) as endogenous antioxidant molecules, and catalase (CAT), were measured in all blood samples [25]. 

The TAC was assessed using the protocol described by Veskoukis et al. [26]. Ιn brief, 20 μL of each plasma sample was mixed with 10 mM sodium phosphate buffer (pH = 7.4; 480 μL), and 0.1 mM 2,2-diphenyl-1-picrylhydrazyl radical (DPPH^•^) solution (500 μL), the mixture remained in an incubator in the dark for 60 min at room temperature (RT), centrifuged (15,000× *g*, 3 min, 4 °C), and the optical density was measured at 520 nm. The TAC was calculated on the basis of the mmol DPPH^•^ reduced by the antioxidants present in the blood. 

TBARS were assessed using the protocol described by Veskoukis et al. [27]. For the assay, 100 μL of plasma was mixed with 35% trichloroacetic acid (TCA) (500 μL) and 200 mM Tris-HCl pH = 7.4 (500 μL), the mixture remained in an incubator for 10 min at RT, and 1 mL of 2 M Νa_2_SO_4_ and 55 mM of thiobarbituric acid (TΒA) was added. Following a 45 min incubation at 95 °C, 1 mL of 70% TCA was added to the mixture, and then the samples were centrifuged (11,200× *g*, 3 min, 20 °C) and the optical density was measured at 530 nm. The concentration of TBARS was calculated on the basis of the millimolar extinction coefficient of malonyldialdehyde (MDA) (156 l/mmol/cm). 

The GSH was assessed using the protocol described by Veskoukis et al. [28]. Briefly, 20 μL of erythrocyte lysate treated with TCA was mixed with 67 mM phosphate buffer (pH = 7.95; 660 μL) and 1 mM 5. 5-dithiobis (2 nitrobenzoic acid) (DTΝB) (30 μL). The mixture remained in an incubator for 15 min in the dark at RT and the optical density was measured at 412 nm. The GSH concentration was calculated on the basis of the millimolar extinction coefficient of DTNΒ (13.6 l/mmol/cm). 

Catalase activity was determined in the erythrocyte lysate. In brief, 4 μL of erythrocyte lysate (dilution of 1:10) was added to 2991 μL of 67 mmol/L sodium potassium phosphate (pH 7.4), and the samples remained in an incubator for 10 min at 37 °C. A total of 5 μL of 30% hydrogen peroxide was added to the mixture, and the change in optical density was immediately read at 240 nm for 2 min. The calculation of catalase activity was based on the molar extinction coefficient of H_2_O_2_ [29].

The hemoglobin concentration of erythrocyte lysate was calculated using a commercially available kit (Dutch Diagnostics, Ζutphen, Holland) since the GSH and CAT results were expressed as μmol/g Hb and U/mg Hb, respectively. All measurements were performed in triplicate. Ιn all cases, standard reagents were used (Sigma-Aldrich, Saint Louis, MO, USA).

### 2.4. Data Analysis

The data were analyzed using the statistical program JASP 16.1. The normality of the data distribution was assessed with the Shapiro–Wilk test and the homogeneity of variances was evaluated with the Levene test. Repeated measures ANOVA was run to evaluate the effect of the sampling day (day) of the vaccines and Vit E/Se administration (group), and their interactions (group x day) on the oxidative stress indicators evaluated, as well as to assess the significance of their differences among groups within sampling days and among days within each group. Post-hoc comparisons were done with a Tukey test. A value of *p* ≤ 0.05 was considered significant in all comparisons, and the data are expressed as the mean ± sem.

## 3. Results

After vaccination, no local irritation at the injection site was observed and all animals remained clinically healthy, with the rectal temperatures within reference interval (39–40 °C) throughout the observation period.

As it is shown in Table 1, the administration of the vaccines and Vit E/Se (group) had a significant effect on the TAC and GSH but not on the TBARS and CAT. However, all redox biomarkers evaluated were significantly affected by the day of sampling and the group × day interaction except for CAT, which was unaffected by the group × day. The fluctuations observed in their serum concentrations throughout the study are presented in the Supplementary Materials (Figures S1–S4).

Marginal TAC mean values (Table 2) were significantly lower in groups V and VE compared to the control and group E (*p* < 0.05; Table 2), and significantly lower on days 6, 14, and 21 than at 0 h (*p* < 0.05; Table 2). Within the control group and in group E, the mean TAC values at all sampling points were not significantly different than the baseline values recorded at the onset of the study (0 h; *p* > 0.05). Within group V, the mean TAC values were significantly lower on sampling days 2, 10, 14, and 21 compared to 0 h (*p* < 0.05). Within the VE group, the mean TAC was significantly higher on D1 and significantly lower on D10 than at 0 h (*p* < 0.05). In addition, on D2 and D10, the TAC values in groups V and VE were significantly lower than in the control and E groups (*p* < 0.05). On D14, the TAC in group V was also significantly lower compared to the control and E group, whereas the TAC in group VE was significantly lower only than that in the control group (*p* < 0.05).

The marginal mean GSH concentration (Table 3) was significantly higher in the E group compared to the controls (*p* < 0.05), whereas no significant differences were detected among the other groups (*p* > 0.05). The marginal mean GSH was also significantly higher on days 1, 2, 6, and 21 compared to 0 h (*p* < 0.05). On day 1, the mean GSH in group E was significantly higher than all the other groups, and on day 14, it was significantly higher in group VE than in the control and E groups (*p* < 0.05; Table 3). Within the control group, the mean GSH remained practically stable among the sampling points, and no significant difference was detected (*p* > 0.05; Table 3). Within the E group, the mean GSH on day 1 was significantly higher than all the other sampling points, and on day 2, it was significantly higher compared to 0 h (*p* < 0.05; Table 2). Within the V group, the mean GSH was significantly higher on day 21 than at 0 h, whereas in the VE group, a significantly higher value was recorded on day 14 compared to 0 h (*p* < 0.05; Table 2). 

TBARS were significantly reduced at 12 h compared to 0 h and then significantly increased on day 1, remaining significantly higher than at 12 h until the end of the study period (*p* < 0.05; Table 4). Within the control group, the mean TBARS were not significantly different among the sampling points, although a numerical reduction was also observed at 12 h compared to 0 h (*p* > 0.05; Table 4). A similar numerical reduction was also observed within the E group, and the mean TBARS values were significantly higher on day 10 compared to 12 h (*p* < 0.05; Table 4). The reduction in the mean TBARS values between 0 h and 12 h was significant within the V and VE groups. Within the V group, the TBARS concentration was then significantly increased on day 1 and remained significantly higher than at 12 h until the end of the study period (*p* < 0.05; Table 4), with exception of days 4 and 10, where numerically higher values than at 12 h (*p* > 0.05) were recorded. Within the VE group, the mean TBARS were significantly increased on day 2 compared to 12 h (*p* < 0.05; Table 4), but the values recorded on the following sampling days were not significantly different than at 12 h (*p* > 0.05; Table 4).

Marginal mean CAT concentration was significantly higher on 12 h and on days 10 and 14 compared to 0 h (*p* < 0.05; Table 5). On day 14, higher CAT values than at 0 h were detected within all groups, but the difference was significant only within the VE group (*p* < 0.05; Table 5). Among the groups, no significant difference was noted at any time point (*p* > 0.05; Table 5).

## 4. Discussion

To the best of our knowledge, this is the first study designed to assess the redox status at repeated intervals over a short time period of 21 days after vaccination in pregnant ewes. It was selected to evaluate the redox status after the annual booster vaccination because it is applied in all animals of a flock and, in fact, in animals at a crucial productive stage when the generation of ROS is abundant. The sampling period was set at 21 days to include, at least, the minimum time period of 2 weeks, during which the immune system is activated after the entry of an antigen to produce antigen-specific antibodies. Concerning the frequency of blood samplings, since there is no such guide in the available literature and considering the acute stress caused by the injection of the vaccine itself, it was determined that we try to evaluate the redox biomarkers at very short intervals close to vaccination and at wider intervals in the following weeks. In most previous studies, the oxidant–antioxidant balance following active immunization has been evaluated at longer intervals, i.e., at 2 weeks or longer after vaccination [14,15,17,30,31]. The current results show a decrease in the total antioxidant capacity of vaccinated ewes compared to non-vaccinated ones. However, this study did not reveal significant oxidative stress after the annual booster vaccination of pregnant ewes with a polyvalent clostridial vaccine, as indicated by the low levels of TBARS, a lipid peroxidation index. Notably, a clear antioxidant action of the Vit E/Se administration was recorded only in the ewes of group E, where no microbial trigger was imposed on the ewes. The antioxidant effect of Vit E supplementation is dose- and route-dependent [32]. The administration of Vit E leads to an increase in Vit E in serum in a time- and dose-dependent manner, showing a rapid elevation by the 8th hour after a single intramuscular injection and, thereafter, a rapid decline [33]. 

The measurement of the total antioxidant capacity (TAC) constitutes a non-specific biomarker for evaluating the effect of a treatment on redox status, as it may describe the dynamic balance between pro-oxidants and antioxidants in an animal’s blood circulation when baseline values do not exist [34]. In a recent study performed on sheep vaccinated with Rev.1 [14], the total antioxidant status was decreased one month after vaccination. In the present study, the TAC showed the lowest levels in vaccinated ewes at 2 and 10days following vaccination in both groups V and VE compared to groups C and E (*p* < 0.05) and remained at low levels 14 days after vaccination in only group V compared to the other three groups. The latter may be attributed to the antioxidant effect of Vit E/Se administration in the vaccinated ewes of group VE. Accordingly, the total antioxidant capacity was found to be decreased in cattle 28 days after vaccination with tick recombinant antigens [16]. However, in the present study, on day 21, the TAC levels were normalized among the groups, which may be related to the different vaccines used and the fact that the time of delivery was approaching. 

Based on the present results, the highest levels of GSH were recorded in group E at 24 h after the injection of Vit E/Se; thereafter, the GSH levels remained higher than in the other three groups until day 4. This increase in the GSH levels 24 h after Vit E/Se administration in group E might be attributed to the fact that Vit E acts as a scavenger itself. On the other hand, the biological effects of selenium are mainly due to its incorporation into selenoproteins, particularly into the glutathione peroxidase enzyme (GPx), which removes potentially damaging lipid hydro-peroxides and hydrogen peroxides and protects the immune cells from oxidative stress-induced damage [35]. Given the extensive number of selenoproteins, there are mechanisms that regulate the priority of selenium use according to the needs of the body [6]. This hierarchy affects not only the utilization of selenium within the same tissue but also selenium retention in other tissues, leading to differences in the antioxidant capacity of each tissue [6]. Accordingly, the supplementation of Vit E and Se in pregnant ewes one month before lambing [36] and in dairy cows during the late stages of pregnancy [37] induced an increase in blood GPx activity. Likewise, in male vaccinated lambs, the dietary supplementation of organic or inorganic Se improved the GPx activity from the 30th day after vaccination [31]. Vitamin E (alpha-tocopherol) is the main liposoluble antioxidant present in cellular membranes [38]. It has the capacity to quench reactive oxygen species, thus decreasing the formation of peroxides that can hamper the normal function of neutrophils and macrophages [39,40]. Thus, this vitamin protects the lipid membrane, receptors, and other cellular components involved in modulating the immune response [8,41]. The latter can explain the elevation in GSH levels 14 days post-vaccination in the VE group, the period when the antibody titers are expected to increase [42]. Contrary to the present GSH results, in *Brucella*-vaccinated pregnant ewes, GSH levels remained low at 2 and 4 weeks and increased only after the 8th week post-vaccination [15], while in *Brucella*-vaccinated and -challenged goats, GSH levels remained low 14 and 28 days after vaccination and only recovered 60 days after the challenge [31]; this result was attributed to the higher levels of glutathione-S-transferase (GST). According to the latter study, CAT values peaked 28 days after vaccination or challenge with Brucella, which is indicative of the antioxidant effect against lipid peroxidation. In a study with goats infected with *Anaplasma spp* [43], a significant decrease in lipid peroxidation and an increase in CAT were found 10 days after the administration of Vit E/Se, but no difference was detected in the GSH values. According to our study, CAT activity was significantly increased 14 days post-vaccination in the VE group, which is in accordance with the aforementioned study and also may be related to the different vaccines used and the reduction of oxidative stress, which means that all peri-parturient ewes had a low antioxidant capacity [44].

The redox status during the immune response could be affected by various factors, i.e., species, age, microbial agent, and active or passive route. In a recent study conducted by Contreras et al. [16], no lipid peroxidation was found during the active immunization, something that could be assumed for the interpretation of the TBARS results of the present study. The TBARS showed a significant decline 12 h after the treatment within both the VE and V groups. In a study conducted on newborn calves, the administration of Vit E resulted in a decrease in TBARS at 12 and 24 h after injection or oral administration [32]. A reduction in TBARS was also observed in experimentally parasitized sheep receiving food supplemented with Se or Vit E/Se [8]. However, others [15] recorded higher malondialdehyde levels in pregnant ewes vaccinated against Brucellosis at 4 or 8 weeks post-vaccination depending on the route of administration. The interpretation of the present results cannot be compared to previous studies since different protocols and vaccines in different species and/or ages were studied. Despite the observed within-group variations, the TBARS were not significantly different among groups either in total or at each sampling point in the present study. This indicates that vaccination against clostridial diseases is not associated with significant lipid peroxidation. It could probably be attributed to the fact that the animals had already been immunized more than once before with the same vaccine. In addition, the ewes were adequately fed a balanced ration containing the required vitamins, trace minerals, and macroelements for dry ewes that may have supported the antioxidant defense system to cope with the oxidative stress caused by the active immunization a few weeks before parturition.

## 5. Conclusions

In the context of this study, it can be concluded that annual booster immunization against clostridial diseases leads to a decrease in the total antioxidant capacity, but it is not associated with significant oxidative stress in pregnant ewes in the last month before the expected day of lambing. Of course, further studies are necessary in order to investigate whether these vaccines have the same effect in first-time immunized animals and if vaccination against other diseases in this period affects the oxidant/antioxidant balance.

## Figures and Tables

**Table 1:** Effects (*p* values) of group, day of sampling (day), and their interactions on the concentrations of the selected redox biomarkers evaluated (TAC, GSH, TBARS, and CAT).

Effect	Redox Biomarkers			
	TAC	GSH	TBARS	CAT
Group	<0.001	0.042	0.153	0.670
Day	<0.001	<0.001	<0.001	<0.001
Group × day	<0.001	<0.001	0.004	0.093

**Table 2:** Post-hoc comparisons of marginal mean ± SE values of TAC (mmol DPPH/L) throughout the study period among groups and sampling days, and post-hoc comparison of mean ± SE TAC values obtained at each sampling among sampling days within each group and among groups within sampling days.

Time Relative to Administrations	Group				Marginal Sampling Day Mean
	Control	E	V	VE	
0 h	0.61 ± 0.03 ^aA^	0.47 ± 0.05 ^abcA^	0.60 ± 0.01 ^bA^	0.45 ± 0.04 ^bcA^	0.52 ± 0.03 ^cd^
12 h	0.51 ± 0.04 ^aA^	0.58 ± 0.04 ^bcA^	0.42 ± 0.07 ^abA^	0.50 ± 0.11 ^bcA^	0.51 ± 0.03 ^cd^
D1	0.55 ± 0.04 ^aA^	0.59 ± 0.05 ^bcA^	0.59 ± 0.03 ^bA^	0.67 ± 0.03 ^cA^	0.59 ± 0.03 ^d^
D2	0.67 ± 0.05 ^aA^	0.63 ± 0.15 ^cA^	0.24 ± 0.04 ^aB^	0.33 ± 0.01 ^abB^	0.46 ± 0.03 ^bc^
D4	0.46 ± 0.01 ^aA^	0.55 ± 0.03 ^bcA^	0.41 ± 0.02 ^abA^	0.39 ± 0.03 ^bA^	0.44 ± 0.03 ^bc^
D6	0.41 ± 0.04 ^aA^	0.33 ± 0.02 ^aA^	0.35 ± 0.03 ^abA^	0.41 ± 0.04 ^bA^	0.37 ± 0.03 ^ab^
D10	0.59 ± 0.05 ^aA^	0.57 ± 0.03 ^bcA^	0.20 ± 0.05 ^aB^	0.14 ± 0.02 ^aB^	0.37 ± 0.03 ^ab^
D14	0.69 ± 0.03 ^aA^	0.56 ± 0.03 ^bcAC^	0.28 ± 0.02 ^aB^	0.33 ± 0.03 ^abC^	0.45 ± 0.03 ^bc^
D21	0.35 ± 0.04 ^aA^	0.32 ± 0.04 ^aA^	0.26 ± 0.03 ^aA^	0.34 ± 0.02 ^abA^	0.31 ± 0.03 ^a^
Marginal group mean	0.53 ± 0.02 ^A^	0.50 ± 0.02 ^A^	0.37 ± 0.02 ^B^	0.41 ± 0.02 ^B^	

A,B,C—Different superscripts in the same row denote significant difference (*p* < 0.05). a,b,c,d—Different superscripts in the same column denote significant difference (*p* < 0.05). 0 h: pre-treatment; 12 h: 12 h post-treatment; D1 to D21: days 1 to 21 post-treatment.

**Table 3:** Post-hoc comparisons of marginal mean ± SE values of GSH (μmol/gr Hb) throughout the study period among groups and sampling days, and post-hoc comparison of mean ± SE GSH values obtained at each sampling among sampling days within each group and among groups within sampling days.

Time Relative to Administrations	Group				Marginal Sampling Day Mean
	Control	E	V	VE	
0 h	1.59 ± 0.35 ^aA^	1.75 ± 0.09 ^aA^	2.34 ± 0.37 ^abA^	2.08 ± 0.37 ^aA^	2.02 ± 0.23 ^ab^
12 h	1.26 ± 0.23 ^aA^	2.26 ± 0.21 ^aA^	1.61 ± 0.28 ^aA^	2.06 ± 0.37 ^aA^	1.87 ± 0.23 ^a^
D1	3.58 ± 0.30 ^aA^	9.40 ± 1.18 ^cB^	3.88 ± 0.46 ^abcA^	3.46 ± 0.76 ^abA^	5.17 ± 0.23 ^e^
D2	3.73 ± 0.63 ^aA^	5.60 ± 0.30 ^bA^	3.64 ± 0.39 ^abcA^	4.18 ± 0.48 ^abA^	4.36 ± 0.23 ^de^
D4	2.19 ± 0.51 ^aA^	3.28 ± 0.39 ^abA^	2.66 ± 0.18 ^abcA^	2.68 ± 0.52 ^abA^	2.78 ± 0.23 ^ab^
D6	1.69 ± 0.27 ^aA^	3.00 ± 0.20 ^aA^	4.10 ± 0.46 ^bcA^	3.99 ± 0.74 ^abA^	3.27 ± 0.23 ^c^
D10	2.30 ± 0.78 ^aA^	3.20 ± 0.36 ^aA^	2.93 ± 0.59 ^abcA^	3.91 ± 0.44 ^abA^	3.16 ± 0.23 ^bc^
D14	1.15 ± 0.23 ^aA^	1.66 ± 0.30 ^aA^	3.48 ± 0.65 ^abcAB^	5.02 ± 0.58 ^bB^	2.90 ± 0.23 ^b^
D21	2.86 ± 0.70 ^aA^	3.53 ± 0.30 ^abA^	4.81 ± 0.44 ^cA^	4.14 ± 0.59 ^abA^	3.91 ± 0.23 ^cd^
Marginal group mean	2.47 ± 0.34 ^A^	3.82 ± 0.31 ^B^	3.35 ± 0.31 ^AB^	3.58 ± 0.31 ^AB^	

^A,B^ Different superscripts at the same row denote significant difference (*p* < 0.05).^a,b,c,d,e^ Different superscripts at the same column denote significant difference (*p* < 0.05). 0 h: pre-treatment; 12 h: 12 h post-treatment; D1 to D21: days 1 to 21 post-treatment.

**Table 4:** Post-hoc comparisons of marginal mean ± SE values of TBARS (μmol/L) throughout the study period among groups and sampling days, and post-hoc comparison of mean ± SE TBARS values obtained at each sampling among sampling days within each group and among groups within sampling days.

Time Relative to Administrations	Group				Marginal Sampling Day Mean
	Control	E	V	VE	
0 h	5.24 ± 0.53 ^aA^	4.24 ± 0.81 ^abA^	4.55 ± 0.75 ^bA^	6.08 ± 0.26 ^bA^	5.05 ± 0.28 ^cd^
12 h	2.93 ± 0.31 ^aA^	2.27 ± 0.23 ^aA^	1.54 ± 0.13 ^aA^	3.05 ± 0.79 ^aA^	2.47 ± 0.28 ^a^
D1	3.18 ± 0.40 ^aA^	3.76 ± 0.56 ^abA^	4.87 ± 0.98 ^bA^	4.75 ± 0.52 ^abA^	4.16 ± 0.28 ^bcd^
D2	3.80 ± 0.26 ^aA^	5.03 ± 0.65 ^abA^	5.38 ± 0.28 ^bA^	6.16 ± 0.92 ^bA^	5.12 ± 0.28 ^d^
D4	4.00 ± 0.39 ^aA^	4.21 ± 0.56 ^abA^	3.74 ± 0.99 ^abA^	3.43 ± 0.32 ^abA^	3.86 ± 0.28 ^bc^
D6	4.12 ± 0.41 ^aA^	3.13 ± 0.23 ^abA^	4.60 ± 0.21 ^bA^	5.96 ± 0.73 ^abA^	4.48 ± 0.28 ^bcd^
D10	4.87 ± 0.52 ^aA^	5.27 ± 0.57 ^bA^	3.63 ± 0.14 ^abA^	3.94 ± 0.43 ^abA^	4.45 ± 0.28 ^bcd^
D14	4.57 ± 0.72 ^aA^	3.73 ± 0.23 ^abA^	4.75 ± 0.29 ^bA^	4.31 ± 0.12 ^abA^	4.36 ± 0.28 ^bcd^
D21	2.81 ± 0.26 ^aA^	3.16 ± 0.26 ^abA^	4.83 ± 0.35 ^bA^	4.27 ± 0.23 ^abA^	3.79 ± 0.28 ^b^
Marginal group mean	3.97 ± 0.20 ^A^	3.89 ± 0.20 ^A^	4.23 ± 0.20 ^A^	4.68 ± 0.20 ^A^	

^A^ Same superscript in the same row denotes non-significant difference (*p* > 0.05).^a,b,c,d^ Different superscripts in the same column denote significant difference (*p* < 0.05). 0 h: pre-treatment; 12 h: 12 h post-treatment; D1 to D21: days 1 to 21 post-treatment.

**Table 5:** Post-hoc comparisons of marginal mean ± SE values of CAT (U/mg Hb) throughout the study period among groups and sampling days, and post-hoc comparison of mean ± SE CAT values obtained at each sampling among sampling days within each group and among groups within sampling days.

Time Relative to Administrations	Group				Marginal Sampling Day Mean
	Control	E	V	VE	
0 h	32.04 ± 2.33 ^abA^	38.43 ± 1.69 ^abA^	37.86 ± 4.15 ^abA^	34.97 ± 3.06 ^abA^	35.86 ± 2.15 ^ab^
12 h	41.83 ± 2.46 ^abA^	52.23 ± 3.28 ^abA^	40.29 ± 3.52 ^abA^	48.41 ± 5.51 ^abA^	45.72 ± 2.15 ^cd^
D1	43.68 ± 4.85 ^abA^	42.13 ± 4.62 ^abA^	36.96 ± 3.25 ^abA^	41.07 ± 4.63 ^abA^	40.99 ± 2.15 ^abc^
D2	49.95 ± 3.27 ^abA^	42.85 ± 2.70 ^abA^	35.45 ± 2.23 ^aA^	39.67 ± 1.87 ^abA^	42.01 ± 2.15 ^abc^
D4	38.54 ± 2.23 ^abA^	38.83 ± 2.49 ^abA^	40.83 ± 6.55 ^abA^	28.07 ± 2.32 ^aA^	36.60 ± 2.15 ^abc^
D6	27.70 ± 0.67 ^aA^	31.68 ± 3.25 ^aA^	39.89 ± 2.28 ^abA^	35.41 ± 4.51 ^abA^	33.70 ± 2.15 ^a^
D10	48.85 ± 8.13 ^abA^	46.31 ± 3.19 ^abA^	54.11 ± 6.72 ^abA^	51.77 ± 3.82 ^bcA^	50.29 ± 2.15 ^d^
D14	58.32 ± 5.87 ^bA^	69.74 ± 4.26 ^bA^	61.44 ± 7.15 ^bA^	63.29 ± 4.11 ^cA^	63.23 ± 2.15 ^e^
D21	48.27 ± 6.88 ^abA^	48.57 ± 3.24 ^abA^	38.09 ± 4.19 ^abA^	41.33 ± 4.80 ^abA^	44.09 ± 2.15 ^abcd^
Marginal group mean	43.27 ± 2.19 ^A^	45.67 ± 2.19 ^A^	42.8 ± 2.19 ^A^	42.70 ± 2.19 ^A^	

^A^ Same superscript in the same row denotes non-significant difference (*p* > 0.05). ^a,b,c,d,e^ Different superscripts in the same column denote significant difference (*p* < 0.05). 0 h: pre-treatment; 12 h: 12 h post-treatment; D1 to D21: days 1 to 21 post-treatment.

## Data Availability

The corresponding author can provide the data that support the results of the present study upon reasonable request. The data are not publicly available as they form part of the MSc thesis of the first author—in the context of the MSc program “Applications of Molecular Biology, Genetics, Diagnostic Biomarkers” at the Department of Biochemistry and Biotechnology of the University of Thessaly, —and it has not yet been examined, approved, or uploaded in the official depository of Masters’ Theses of the University of Thessaly.

## Supplementary Materials

The following supporting information can be downloaded at: https://www.mdpi.com/article/10.3390/vaccines10060898/s1, Figure S1: Mean TAC values (mmol DPPH/L) detected in all groups of ewes throughout the study period; Figure S2: Mean GSH values (μmol/gr Hb) detected in all groups of ewes throughout the study period; Figure S3: Mean TBARS values (μmol/L) detected in all groups of ewes throughout the study period; Figure S4: Mean CAT values (U/mg Hb) detected in all groups of ewes throughout the study period.

## References

[1] Knight J.A. (2000). Review: Free radicals, antioxidants, and the immune system. Ann. Clin. Lab. Sci..

[2] Sies H. (2015). Oxidative stress: A concept in redox biology and medicine. Redox Biol..

[3] Sharifi-Rad M., Anil Kumar N.V., Zucca P., Varoni E.M., Dini L., Panzarini E., Rajkovic J., Tsouh Fokou P.V., Azzini E., Peluso I. (2020). Lifestyle, Oxidative Stress, and Antioxidants: Back and Forth in the Pathophysiology of Chronic Diseases. Front. Physiol..

[4] Lu S.C. (2013). Glutathione synthesis. Biochim. Biophys. Acta.

[5] Evans P., Halliwell B. (2001). Micronutrients: Oxidant/antioxidant status. Br. J. Nutr..

[6] Rooke J.A., Robinson J.J., Arthur J.R. (2004). Effects of vitamin E and selenium on the performance and immune status of ewes and lambs. J. Agric. Food Chem..

[7] Forman H.J., Zhang H., Rinna A. (2009). Glutathione: Overview of its protective roles, measurement, and biosynthesis. Mol. Asp. Med..

[8] Leal M.L., de Camargo E.V., Ross D.H., Molento M.B., Lopes S.T., da Rocha J.B. (2010). Effect of selenium and vitamin E on oxidative stress in lambs experimentally infected with Haemonchus contortus. Vet. Res. Commun..

[9] Lacasta D., Ferrer L.M., Ramos J.J., González J.M., Ortín A., Fthenakis G.C. (2015). Vaccination schedules in small ruminant farms. Vet. Microbiol..

[10] Fernandez-Miyakawa M.E., Redondo L.M., Gopalakrishnakone P., Stiles B., Alape-Girón A., Dubreuil J.D., Mandal M. (2016). Role of Clostridium perfringens Alpha, Beta, Epsilon and Iota toxins in Enterotoxemia of monogastrics and Ruminants. Microbial Toxins.

[11] Fthenakis G.C., Arsenos G., Brozos C., Fragkou I.A., Giadinis N.D., Giannenas I., Mavrogianni V.S., Papadopoulos E., Valasi I. (2012). Health management of ewes during pregnancy. Anim. Reprod. Sci..

[12] Garrel C., Fowler P.A., Al-Gubory K.H. (2010). Developmental changes in antioxidant enzymatic defences against oxidative stress in sheep placentomes. J. Endocrinol..

[13] Spears J.W., Weiss W.P. (2008). Role of antioxidants and trace elements in health and immunity of transition dairy cows. Vet. J..

[14] Çiftci G., Çiftci A. (2021). The assessment of the protein profiles and oxidant/antioxidat status in conjunctival Brucella melitensis Rev1 vaccinated sheep. Etlik Vet. Mikrobiyoloji Derg..

[15] Al-Khafaji W., Al-Farwachi M. (2012). Antioxidant status in pregnant ewes vaccinated with Rev 1 against brucellosis. Iraqi J. Vet. Sci..

[16] Contreras M., Peres Rubio C., de la Fuente J., Villar M., Merino O., Mosqueda J., Ceron J.J. (2020). Changes in Serum Biomarkers of Oxidative Stress in Cattle Vaccinated with Tick Recombinant Antigens: A Pilot Study. Vaccines.

[17] Anugu S., Petersson-Wolfe C.S., Combs G.F., Petersson K.H. (2013). Effect of vitamin E on the immune system of ewes during late pregnancy and lactation. Small Rumin. Res..

[18] Tengerdy R.P., Meyer D.L., Lauerman L.H., Lueker D.C., Nockels C.F. (1983). Vitamin E-enhanced humoral antibody response to Clostridium perfringens type D in sheep. Br. Vet. J..

[19] Giadinis N., Koptopoulos G., Roubles N., Siarkou V., Papasteriades A. (2000). Selenium and vitamin E effect on antibody production of sheep vaccinated against enzootic abortion (Chlamydia psittaci). Comp. Immunol. Microbiol. Infect. Dis..

[20] Kumar N., Garg A.K., Dass R.S., Chaturvedi V.K., Mudgal V., Varshney V.P. (2009). Selenium supplementation influences growth performance, antioxidant status and immune response in lambs. Anim. Feed. Sci. Technol..

[21] Daniels J.T., Hatfield P.G., Burgess D.E., Kottt R.W., Bowman J.G. (2000). Evaluation of ewe and lamb immune response when ewes were supplemented with vitamin E. J. Anim. Sci..

[22] Hatfield P.G., Robinson B.L., Minikhiem D.L., Kott R.W., Roth N.I., Daniels J.T., Swenson C.K. (2002). Serum alpha-tocopherol and immune function in yearling ewes supplemented with zinc and vitamin E. J. Anim. Sci..

[23] NRC (2007). Nutrient Requirements of Small Ruminants.

[24] Dønnem I., Randby Å.T., Hektoen L., Avdem F., Meling S., Våge Å.Ø., Ådnøy T., Steinheim G., Waage S. (2015). Effect of vitamin E supplementation to ewes in late pregnancy on the rate of stillborn lambs. Small Rumin. Res..

[25] Veskoukis A.S., Kerasioti E., Priftis A., Kouka P., Spanidis Y., Makri S., Kouretas D. (2019). A battery of translational biomarkers for the assessment of the in vitro and in vivo antioxidant action of plant polyphenolic compounds: The biomarker issue. Curr. Opin. Toxicol..

[26] Veskoukis A.S., Nikolaidis M.G., Kyparos A., Kokkinos D., Nepka C., Barbanis S., Kouretas D. (2008). Effects of xanthine oxidase inhibition on oxidative stress and swimming performance in rats. Appl. Physiol. Nutr. Metab..

[27] Veskoukis A.S., Kyparos A., Nikolaidis M.G., Stagos D., Aligiannis N., Halabalaki M., Chronis K., Goutzourelas N., Skaltsounis L., Kouretas D. (2012). The antioxidant effects of a polyphenol-rich grape pomace extract in vitro do not correspond in vivo using exercise as an oxidant stimulus. Oxidative Med. Cell. Longev..

[28] Veskoukis A.S., Kyparos A., Paschalis V., Nikolaidis M.G. (2016). Spectrophotometric assays for measuring redox biomarkers in blood. Biomark. Biochem. Indic. Expo. Response Susceptibility Chem..

[29] Makri S., Kafantaris I., Stagos D., Chamokeridou T., Petrotos K., Gerasopoulos K., Mpesios A., Goutzourelas N., Kokkas S., Goulas P. (2017). Novel feed including bioactive compounds from winery wastes improved broilers’ redox status in blood and tissues of vital organs. Food Chem. Toxicol. Int. J. Publ. Br. Ind. Biol. Res. Assoc..

[30] Ramos A., Laguna I., de Lucia M.L., Martin-Palomino P., Regodon S., Miguez M.P. (2010). Evolution of oxidative/nitrosative stress biomarkers during an open-field vaccination procedure in sheep: Effect of melatonin. Vet. Immunol. Immunopathol..

[31] Kumar A., Gupta V.K., Verma A.K., Rajesh M., Anu R., Yadav S.K. (2017). Lipid Peroxidation and Antioxidant System in Erythrocytes of Brucella Vaccinated and Challenged Goats. Int. J. Vaccines Vaccin.

[32] Mokhber-Dezfouli M.R., Rahimikia E., Asadi F., Nadalian M.G. (2008). The role of route of vitamin E administration on the plasma antioxidant activity and lipid peroxidation in newborn calves. Basic Clin. Pharmacol. Toxicol..

[33] Njeru C.A., McDowell L.R., Wilkinson N.S., Linda S.B., Williams S.N., Lentz E.L. (1992). Serum alpha-tocopherol concentration in sheep after intramuscular injection of DL-alpha-tocopherol. J. Anim. Sci..

[34] Celi P. (2010). The role of oxidative stress in small ruminants’ health and production. Rev. Bras. Zootec..

[35] Salman S., Khol-Parisini A., Schafft H., Lahrssen-Wiederholt M., Hulan H.W., Dinse D., Zentek J. (2009). The role of dietary selenium in bovine mammary gland health and immune function. Anim. Health Res. Rev..

[36] Morgante M., Beghelli D., Pauselli M., Dall’Ara P., Capuccella M., Ranucci S. (1999). Effect of administration of vitamin E and selenium during the dry period on mammary health and milk cell counts in dairy ewes. J. Dairy Sci..

[37] Lacetera N., Bernabucci U., Ronchi B., Nardone A. (1996). Effects of selenium and vitamin E administration during a late stage of pregnancy on colostrum and milk production in dairy cows, and on passive immunity and growth of their offspring. Am. J. Vet. Res..

[38] Kay M.M., Bosman G.J., Shapiro S.S., Bendich A., Bassel P.S. (1986). Oxidation as a possible mechanism of cellular aging: Vitamin E deficiency causes premature aging and IgG binding to erythrocytes. Proc. Natl. Acad. Sci. USA.

[39] Butterick C.J., Baehner R.L., Boxer L.A., Jersild R.A. (1983). Vitamin E--a selective inhibitor of the NADPH oxidoreductase enzyme system in human granulocytes. Am. J. Pathol..

[40] Sharmanov A.T., Aidarkhanov B.B., Kurmangalinov S.M. (1990). Effect of vitamin E deficiency on oxidative metabolism and antioxidant enzyme activity of macrophages. Ann. Nutr. Metab..

[41] Meydani M. (1995). Vitamin E. Lancet.

[42] Cooper B.S. (1976). The transfer from ewe to lamb of clostridial antibodies. N. Z. Vet. J..

[43] Dhanasree G., Pillai U.N., Deepa C., Ambily V.R., Shynu M., Sunanda C. (2020). Evaluation of oxidative stress in caprine anaplasmosis and effect of vitamin E-selenium in monitoring oxidative stress. Trop. Anim. Health Prod..

[44] Mutinati M., Piccinno M., Roncetti M., Campanile D., Rizzo A., Sciorsci R. (2013). Oxidative stress during pregnancy in the sheep. Reprod. Domest. Anim. Zuchthyg..

