# Supplementary material for: Redox Status of Pregnant Ewes after Vaccination against Clostridial Diseases
Source: Vaccines (Basel). 2022 Jun 5;10(6):898. doi: 10.3390/vaccines10060898 (PMC9229268; doi:10.3390/vaccines10060898)
Supplement: Supplementary file 1 [file vaccines-10-00898-s001.zip › vaccines-1700815-supplementary.pdf]

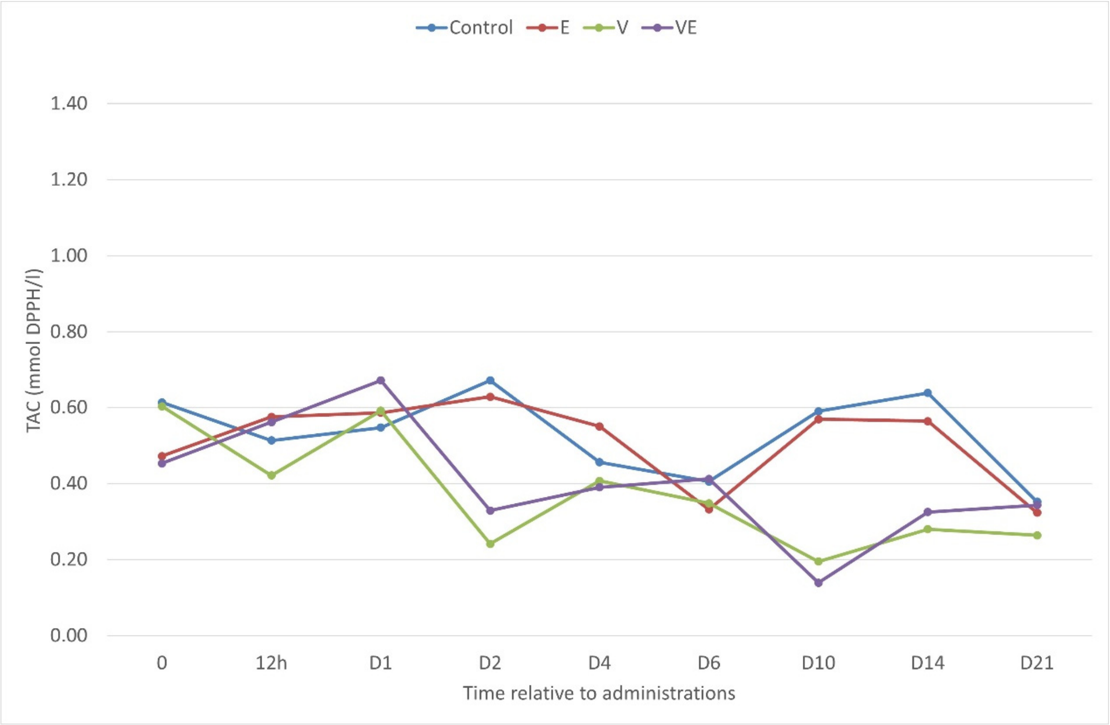

Figure S1. Mean TAC values (mmol DPPH/L) detected in all groups of ewes throughout the study period

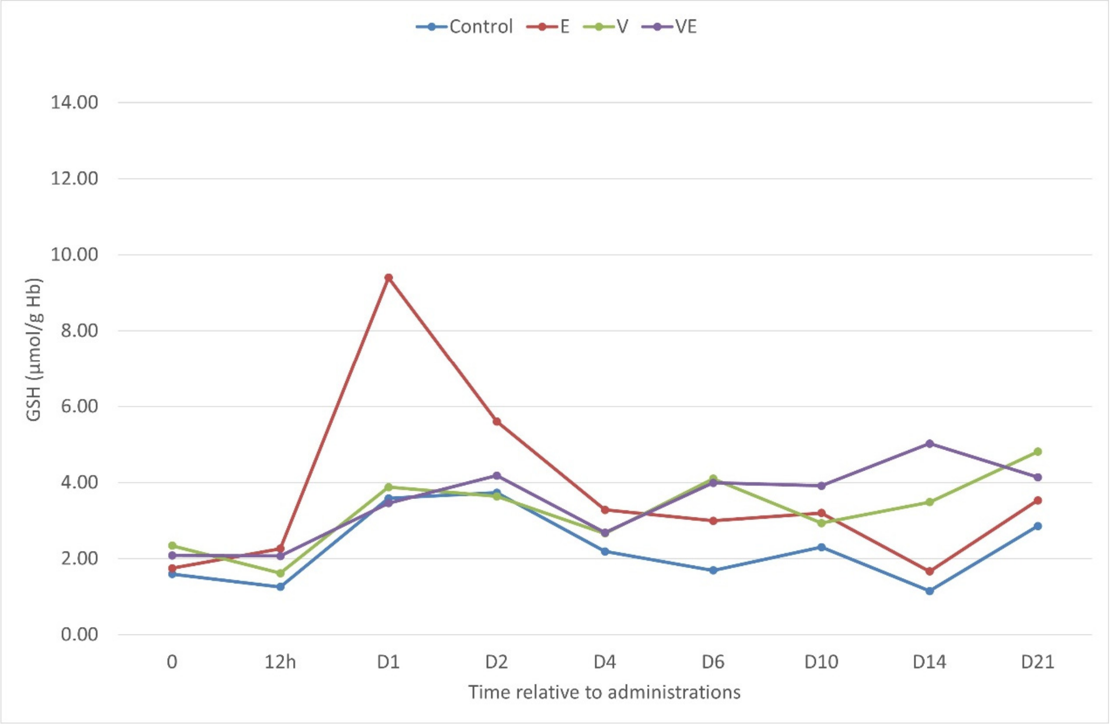

Figure S2. Mean GSH values (μmol/gr Hb) detected in all groups of ewes throughout the study period

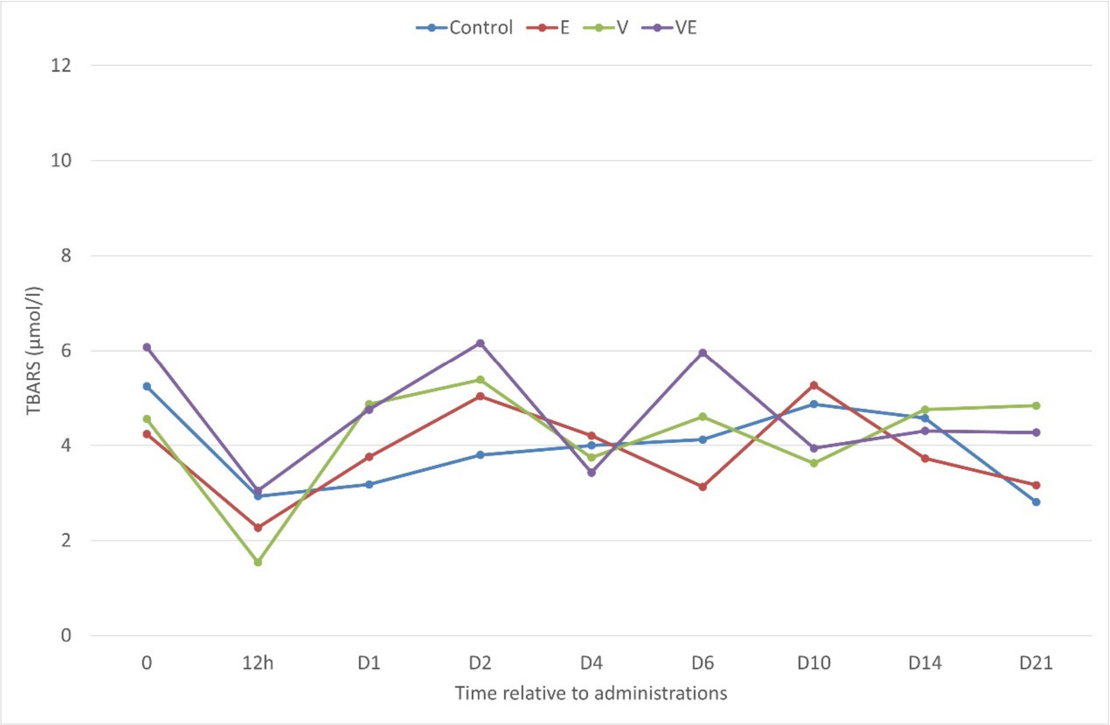

Figure S3. Mean TBARS values (μmol/L) detected in all groups of ewes throughout the study period.

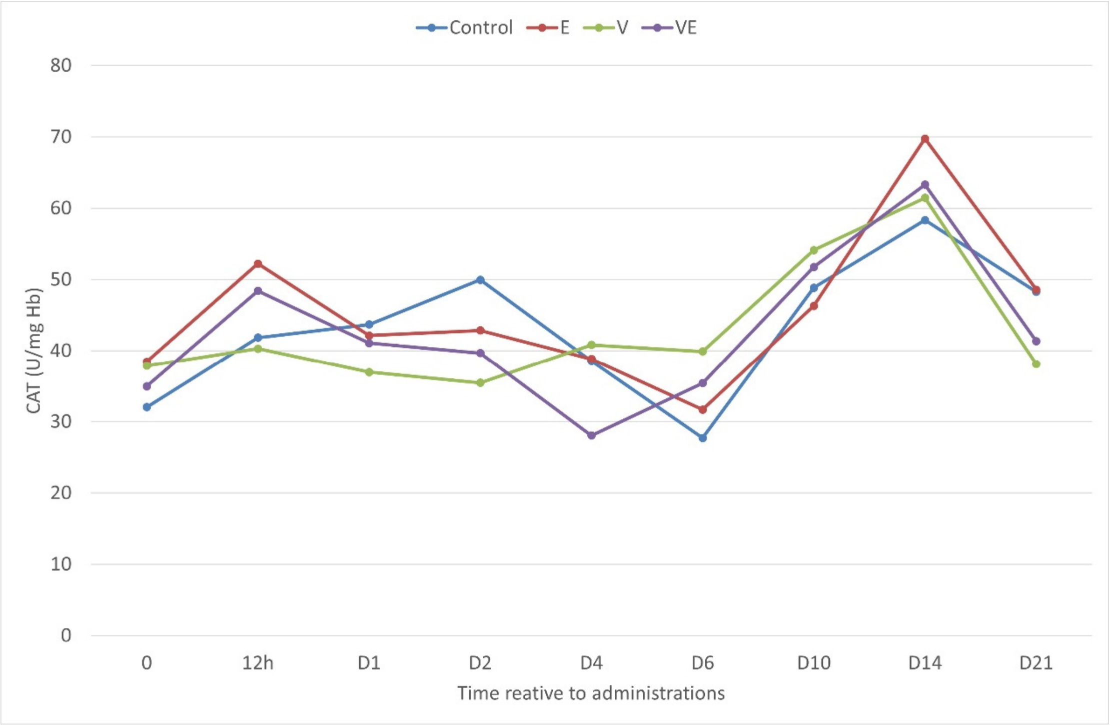

Figure S4. Mean CAT values (U/mg Hb) detected in all groups of ewes throughout the study period
